# Changes in Management, Welfare, Emotional State, and Human-Related Docility in Stallions

**DOI:** 10.3390/ani12212981

**Published:** 2022-10-30

**Authors:** Silvana Popescu, Eva Andrea Lazar, Cristin Borda, Anamaria Blaga Petrean, Elena Mitrănescu

**Affiliations:** 1 Department of Animal Hygiene and Welfare, Faculty of Veterinary Medicine, University of Agricultural Sciences and Veterinary Medicine, 400372 Cluj-Napoca, Romania; 2 Association for the Welfare of Horses, 725700 Vatra Dornei, Romania; 3 Department of Animal Hygiene and Welfare, Faculty of Veterinary Medicine, University of Agronomic Sciences and Veterinary Medicine of Bucharest, 050097 Bucharest, Romania

**Keywords:** stallions, group housing, horse welfare, human-related docility, qualitative behavioral assessment

## Abstract

**Simple Summary:**

Regardless of age, breed, physiological condition, or gender, all horses are social animals, requiring the company of their own kind. Due to their natural instincts to fight each other, adult stallions are often kept in isolation. Several studies have shown the possibility of free group housing and recommended methods to reduce the detrimental effects of limited social contact on their physical and/or mental wellbeing. Nevertheless, the beneficial effect of transitioning from tie-stalls to free housing on adult stallions’ overall welfare has not been researched before. According to our results, in only two weeks after the management change, the studied stallions had significantly better welfare, improving further over time. Additionally, their human-related responses improved, and their docility did not decrease, despite minimal human–animal contact during the study. Although positive emotional states were identified within the study, these did not correlate consistently with the other parameters assessed. Given the findings in this paper and accounting for all precautions required while making such a management change, we conclude that adult stallions can and should be kept in free group housing to provide them with the best possible conditions to support their optimal welfare.

**Abstract:**

Despite an increase in awareness of their essential needs, many stallions continue to be kept in conditions limiting their social interactions and movement. To supplement the studies which highlight the effects of these practices on selected aspects of equine mental and physical wellbeing, we aimed to monitor a group of 32 adult intact stallions during their transition from tethered housing with limited outdoor access to free group housing through the lens of their overall welfare, perceived emotional status, and docility toward humans. Over three visits (before the management change, two weeks, and three months after, respectively), their welfare, qualitative behavior, and docility were assessed. Analysis of the data collected showed an improvement in the stallions’ overall welfare and no decrease in their docility after their group-release, with a constant correlation between these two aspects. The evaluation of their emotional states was less relevant, lacking consistency between the assessments for most of the descriptors used, warranting further research in similar conditions. Although our study covered a relatively short period of time, our results provide encouraging support for stallion owners in deciding on a similar management change for the welfare of their animals.

## 1. Introduction

A widely known definition [1] states that animal welfare is the state of an individual regarding its attempts to cope with its environment. The degree of coping, or adaptation, concerns simultaneously three aspects of welfare: the state of the animal’s body, mind, and the extent to which its nature is satisfied [2]. For domestic animals, including horses, their housing conditions define their close environment, and the implementation of specific management decisions represents a major factor in shaping environmental challenges for the animal. 

The traditional management of adult stallions relies mostly on stabling, with more or less outside access, but in isolation or with limited interaction with other equines [3]. Although this is an efficient way to avoid fighting (and, thus, the occurrence of injuries), unwanted mating, and the spread of diseases, the natural behaviors and social interaction of these animals are sacrificed [4]. Many authors agree that solitary confinement still practiced in adult stallions can lead to stress [5] because it disregards their need to display natural behaviors [6], negatively impacts their mental and physical health, and, consequently, their welfare [7]. The use of tie-stalls that are no longer accepted in many countries [8] is associated with an increased incidence of health problems in stallions, probably due to lack of exercise and prolonged standing on wet and soiled bedding [9]. Although more appropriate compared with the tied system, individual box-housing also has deficiencies, such as social isolation, a significant limitation of normal grazing behavior, and severe lack of exercise, which can trigger abnormal behaviors and health problems in stallions [10]. The majority of adult stallions in Romania, both in state-owned and private facilities, are kept tethered or in individual boxes, with or without paddock access. 

Some authors observed fewer behavioral disorders in horses living in paddocks or on pasture [4,11,12,13,14], although in stallions, this housing system may favor fights which can cause severe lesions and even the death of the animals in some cases [3,4]. More recently, Gehlen et al. [5] concluded, based on an extended literature review, that group-housing of stallions represents the most adequate system if a few characteristics regarding the exercise area (size and design), the composition of the group, and the horses are considered. Several benefits of this management system have been studied in the reviewed papers on different aspects of equine health or behavior, but not on their overall welfare status considered holistically. 

The aim of the present study was to monitor a group of adult intact stallions during their transition from tethered housing with limited outdoor access in individual paddocks to free group housing through the lens of their overall welfare, perceived emotional status, and docility toward humans. Additionally, several correlations between the studied parameters were investigated. 

## 2. Materials and Methods

Disclaimer: This paper presents an observational study of a stallion group in their transition from tethered to free group-housing conditions. All management decisions were planned and implemented solely by the owner of the animals. Although the paper fully describes the sample characteristics and the context, in order to ensure research repeatability, the authors do not encourage or suggest exact replication of the presented conditions to any lay person, especially where thorough supervision of the animals is not possible.

### 2.1. The Stallions and Their Housing Conditions

The study was performed in the spring of 2022 (April–July) in a privately owned group of 32 stallions (aged between 5 and 18 years). Concerning breeds, they were Hutzul horses (*n* = 7), Pure Bred Arabians (*n* = 3), Romanian Draft horses (*n* = 11), Ardennes (*n* = 7), and local horses with unregistered genealogy (*n* = 4). Purchased gradually over the previous five years, the stallions had been housed in a progressively extended wooden barn, chain-tethered on two sides, with periodic access to free movement in the six paddocks next to the barn. These square-shaped outside exercise areas fenced with wooden rails were on flat, rocky terrain, and each had a surface of 100 m^2^. The 2 m space between adjacent paddocks did not allow physical contact between the stallions. 

The stallions’ daily management was performed manually by the owner and two employees. Cleaning the barn, partially replacing the wood shavings and sawdust used as bedding on the compacted earth flooring, and checking the cleanliness of the water buckets mounted on the sides of the feeder boxes were carried out once per day. The stallions were fed four times per day (three meals of hay and one of a mixture of equal amounts of corn, oat, and barley). At these times, the water in the buckets was also replenished as needed. The stallions were hand-led to the paddocks and back to the barn. Most of them were haltered and fitted with a bit, except for two of them, considered the most docile by the owner, which were led only using a rope attached loosely around the base of their necks. Except for five stallions used for work almost every day (forestry, wood hauling), each stallion had access to free exercise for an average of three hours every three days.

This type of management allowed limited social contact between the animals: a certain degree of visual, olfactive, and auditive contact was always possible, especially for stallions tethered next to each other or let out in neighboring paddocks at the same time, but, generally, tactile interaction (physical touch) was not possible. The five stallions used for forestry work were the only exception. Although never unsupervised by humans, during their work (sometimes in pairs) or while resting at the workplace, these animals used to be left close enough so that they could touch each other. For their whole stabled history, the stallions were kept in the same places in the barn and let out in the paddocks in the same order. All the purebred stallions purchased from national stud farms were bought either directly from the breeding category or had been previously in that category. Although the owner used four of them for breeding his own mares, the main reason to keep the stallions intact was the traditional belief that castration causes loss of physical strength and workforce in the animal. 

The large outside paddock (approximately rectangular shaped) where the stallions were released for their management change had an approximate area of 15 ha. Its terrain varied (approximately 70% flat, 15% slopes with less than 5° inclination, and 15% slopes with more than 5° inclination), and was mostly covered by grass (approximately 80% of the land had a natural mountain-grass coverage), with the bare ground (covered by fallen leaves) only under the mixed species trees (conifers and broadleaf trees) which had grown in patches (on a total surface area of around 20% of the land). The paddock was protected by double electric fencing (3 m distance between the two fences), with two wire-rows on wooden posts, and a third three-row fence (1 m distance from the outer electric fence-line) made of only bright-white rope between wooden posts. The water source was a shallow streamlet (50 cm at its deepest and between 1 and 1.5 m wide) crossing the terrain lengthwise in a rock bed, with two purposefully constructed watering places where the stream course had been widened to approximately 10 m^2^ water-eyes (with a depth less than 50 cm, with additional rock consolidation on the margins around). In addition to these drinking places, the whole length of the streamlet was accessible for the stallions to drink from.

The management change was implemented over a few hours in a single day. Three men with whom the stallions were familiar (the owner and two employees) haltered and led by hand three stallions at the same time, taking them in the order they had been tethered inside the barn (one side of the barn, then the other). This way, each stallion was released at the same time with at least one of his previous neighbors. The animals were led from the barn to the paddock at a distance of about 7–8 m from each other. After entering the paddock, the stallions were led to approximately 45–50 m from each other and then released by removing the halters from their heads. Four additional persons were present, equipped with long whips and ropes, ready to intervene in case of any fighting between the stallions. At the request of the owner, none of the researchers were present at the release of the horses. The description of the procedure is based fully and exclusively on the owner’s declaration. 

Before the management change, all the stallions had been unshod and had their hooves trimmed. During the study, hoof trimming was repeated in some of them, as decided by the owner. To avoid fighting as much as possible, the stallions received no additional feeding to supplement grazing. 

All the procedures required by the study were performed with the consent and in the presence of the owner. The welfare assessment protocol’s application was completely non-invasive, and no animal was stressed in order to be studied. 

### 2.2. Animal Assessments

The farm was visited three times during the stallions’ transition from tethered housing to free group keeping. The first assessment was performed a couple of days before the management change, the second two weeks after the stallions were released on pasture in a single group, and the third evaluation three months after the second visit. 

For the welfare assessment of the stallions, five freedoms were explored [9] through 25 mostly animal-based parameters (Table 1), following mainly the protocol described by Popescu et al. [9]. Differences from the protocol included the omission of some parameters, the inclusion of the water cleanliness assessment, and the fact that for the freedom from fear and distress evaluation, the interaction between the horses and their owner was observed without the involvement of an unfamiliar assessor. 

All assessors had attended a previous training exercise in which at least 80% inter- and intra-assessor repeatability was achieved for each parameter of the welfare assessment protocol. The assessors worked in pairs (one assessing, the other writing the results) by rotation. 

At the end of this assessment, the qualitative behavioral assessment (QBA) of the stallions was performed, as described and recommended by the AWIN welfare assessment protocol for horses [16]. This QBA relies on the ability of humans to integrate perceived details of behavior, posture, and context into descriptions of an animal’s body language using 13 descriptors with expressive, emotional connotations (aggressive, alarmed, annoyed, apathetic, at ease, curious, friendly, fearful, happy, look for contact, relaxed, pushy, and uneasy—in this order) to provide directly relevant information to animal welfare as a useful addition to information obtained from quantitative indicators. To apply this tool, the horses were observed from 5 to ten10 meters, without disturbing them, for approximately 30 s to one minute. Then, the observer departed to a quiet spot and scored the list of the descriptors using visual analogue scales (VAS), one for each term. Each VAS is a horizontal line between a left “minimum” and right “maximum” point, with the expressive quality indicated by the term entirely absent in the whole observation period at the minimum and dominant during the whole observation period at the maximum. The intermediate scores ticked with a mark on the line depending on the intensity and duration of a behavior. The measure for each term is the distance in millimeters from the minimum point to the point where the VAS is ticked. 

In order to score the two parameters which evaluated their human-related docility (Table 2), the stallions were observed by an assessor while being handled by their owner. 

### 2.3. Data Processing and Statistical Analysis

As in the work described by Popescu et al. [9], an individual welfare quality score (IWQS) was calculated for each stallion in each of the three evaluations performed by adding up the scores of the welfare assessment parameters. The range of this score was from a theoretical zero to a maximum of 41, showing better welfare quality with the increase in the score. 

To provide qualitative significance to the numerical results of the welfare assessment, each stallion was included in one of the four qualitative welfare classes according to their IWQS: not classified (scores from 0 to 15), acceptable (between 16 and 25), enhanced (from 26 to 35), and excellent (ranging from 36 to 41). The number of stallions in each qualitative welfare class was calculated as a percentage in each of the three assessments.

The calculation of the docility score (DS) was performed by adding up the scores recorded for the two parameters of the human-related docility test by each stallion in each of the assessments. The higher the DS was, the more docile the animal was considered. 

The results were analyzed using SPSS (SPSS version 17, SPSS Inc., Chicago, IL, USA) software. The mean, standard error of the mean, median, minimum, and maximum were calculated as descriptive statistical parameters. The normality distribution of the data was tested by the Kolmogorov–Smirnov test, and Friedman and subsequent Wilcoxon tests were used to evaluate the parameter changes in relation to time. A principal component analysis (PCA), using a correlation matrix and applying no rotation, was conducted on the QBA descriptors. Because the data were not normally distributed, the Spearman rank correlations were used to study the relationships between the assessed parameters. Differences and correlations were considered statistically significant at a *p*-value < 0.05. 

## 3. Results

### 3.1. Welfare Parameters of the Studied Stallions

The results of the welfare assessments were compared between the three evaluations (Table 3), before (assessment 1—A1), and after the stallions’ management change (assessment 2—A2, assessment 3—A3). For the majority of the evaluated parameters, there were significant differences, except for those which investigated the freedom from fear and distress.

### 3.2. Dynamics of Individual Welfare Quality and Docility Scores of the Stallions during Their Management Transition

The IWQS median increased significantly from the first to the second and third evaluations, while the DS had similar values in the three assessments (Table 4). 

**Figure 1:**
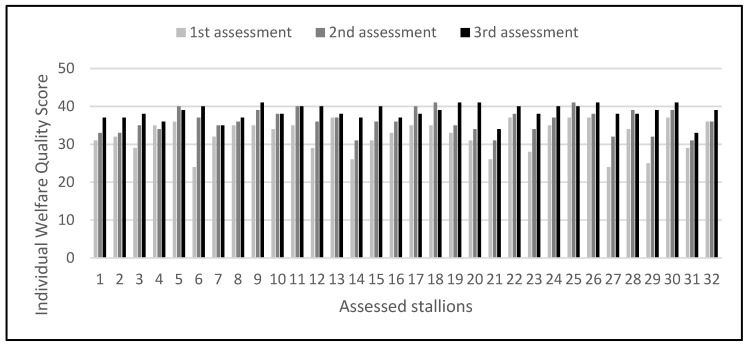
Evolution of the IQWS in the 32 stallions within the three welfare assessments performed.

Without exception, the individual welfare quality scores for the third assessment were higher than those recorded in the first assessment, even where the second assessment score was lower (stallion no. 4) or equal to the first one (stallions no. 13 and 32), or when the second assessment score exceeded (stallions no. 5, 17, 18, 25, and 28) or equaled (stallions no. 7, 10, and 11) the IWQS recorded in the third assessment (Figure 1). 

**Figure 2:**
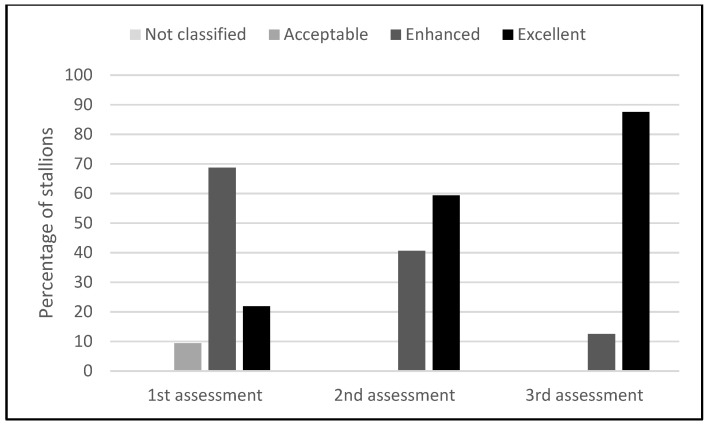
Percentages of stallions in the established qualitative welfare classes (acceptable, enhanced, and excellent) in the three welfare assessments performed.

There were no stallions in the ‘not classified’ qualitative welfare class (according to their IWQS) in any of the assessments. The percentage of stallions included in the other three classes and their variation from one assessment to the other are shown in Figure 2. 

Three stallions were included in the ‘Acceptable’ class in the first assessment but none in the second and third assessments. The rest of the stallions were included in the two superior classes (enhanced and excellent), and their percentage in the ‘Excellent’ class increased from the first to the third assessment (Figure 2). 

For the ‘Acceptable’ class, a statistically significant difference was found between the first and third assessments (*p* = 0.047). The number of stallions in the ‘Enhanced’ class differed significantly between the first and second (*p* = 0.025), between the first and third (*p* < 0.001), and between the second and third assessments (*p* = 0.025). The differences in numbers within the ‘Excellent’ class were significant between the first and third (*p* < 0.001), first and second (*p* = 0.002), and second and third assessments (*p* = 0.011). 

### 3.3. Principal Component Analysis of the Qualitative Behavioral Assessment Descriptors

The PCA was applied to the first and third assessments performed in the stallions before the management change and three and a half months after their release in a single free-housed group. Three principal components have been identified, as shown in Table 5.

In the first assessment, the QBA descriptors on PC1 ranged from negative emotional states, such as fearful and alarmed, to positive, such as friendly and relaxed; on PC2, from pushy to apathetic; on PC3, from apathetic to happy. In the second assessment, the PC1 arranged the QBA descriptors from fearful/alarmed to friendly/relaxed/curious, the PC2 from aggressive to uneasy/pushy, and the PC3 from apathetic to happy. In the third evaluation, the QBA descriptors on PC1 ranged from fearful/alarmed to friendly/curious/relaxed/look for contact, on PC2 from aggressive to pushy, and on PC3 from apathetic to happy. 

Table 6 shows the first three principal components and their coefficients. Generally, on all components, the correlation coefficients have been low (less than 0.2). The strongest correlations were found for the apathy and happiness descriptors (Table 6). 

### 3.4. Correlations between the Assessed Parameters

To explore the relationship between the stallions’ welfare degree, docility, and their emotional states, correlations have been calculated, as shown in Table 7. 

The PC1 correlated significantly with the IWQS in the first (A1) and third (A3) assessments. The PC3 correlated with the IWQS and the DS in the first assessment (A1) and in the second assessment (A2) only with the DS. 

Constantly significant correlations have been found in all three assessments between the stallions’ IWQS and DS (A1, r_s_ = 0.386, *p* = 0.01; A2, r_s_ = 0.357, *p* = 0.05; A3, r_s_ = 0.521, *p* = 0.01).

## 4. Discussion

### 4.1. Dynamics of the Welfare Parameters during the Stallions’ Management Change

According to the results of a recent literature review performed by Gehlen et al. [5], the group husbandry of stallions is not only possible but also a desirable response of horse breeders to the increasing social awareness of animal welfare. There is a considerable body of research relating to the provision of husbandry in such a way as to allow species-specific and natural conditions for all horses, including adult stallions. Several housing systems and transition methods have been tested around the world, more or less traditionally [18], to overcome the popular belief that isolation is an acceptable method for keeping adult stallions safe. 

The transition from tie-stalls to free group housing has generally had a positive influence on the welfare parameters assessed in our study, measurable in as little as two weeks from the implementation of the management change (Table 3). 

#### 4.1.1. Freedom from Hunger and Thirst

One of the important indicators relevant to freedom from hunger and thirst is the body condition score (BCS). Similar to the results reported by Yngvesson et al. [19], no statistically significant differences were found in the BCS of the stallions between the assessment before the management change and the two measurements after it. Nevertheless, in our study, the prevalence of stallions with improper BCS decreased in three months (at the third assessment—A3) of free group housing outdoors. There are many different factors and possible ailments contributing to improper body weight in horses, whether the BCS is too low (thin or emaciated animals) or too high (fat or obese animals). However, in the studied healthy stallions kept in relative inactivity, the access to a more natural lifestyle of outside exercise and grazing seemed to normalize their body weight, promoting fitness in both the formerly overweight and underweight individuals. In a comparative study on breeding horses kept in tie-stalls (stallions) and loose group housing (mares), Sanmartín Sánchez et al. [20] found a higher risk for low BCS in the free-housed mares, despite them having free access to a greater variety of forage than the stallions, in conditions allowing more natural equine behavior. Possible factors of influence on the free-range horses’ BCS highlighted by the mentioned study [20] are lower pasture quality, competition for feeding, and possibly better teeth condition in the stabled stallions. In our study, these factors were not present, and most probably, the fat stallions lost weight because of more physical activity in freedom, and the thin ones ate more, having continuous free access to good quality pasture.

In our study, the husbandry system change also had a positive impact on water cleanliness due to the water source the stallions had access to while free-housed. Water is an essential nutrient for all mammals, especially horses, who need considerable amounts of water for their hindgut microbiota for proper fermentation [21]. Thus, fresh and clean drinking water should be available for horses at all times, not only because of its nutritional importance but also for its role in preventing digestive problems with potentially life-threatening severity. 

#### 4.1.2. Freedom from Discomfort

The hip point assessment is used to reveal possible lesions, present wounds, or healed scars indicative of housing discomfort. Especially in sick horses which spend prolonged periods lying down, the pressure of a bone on a reduced skin surface causes tissular ischemia and subsequent necrosis [20,22]. The hip point (external angle of the ilium) is an anatomical area predisposed to such types of wounds. Similarly, in healthy horses, hip point lesions occur while lying on hard, abrasive surfaces without bedding or when the bedding is insufficient, dirty, or wet [23]. The pre-existent individual conditions that precipitate the occurrence of decubital ulcers include the loss of subcutaneous fat padding in thin equines [22], but also inappropriate hygiene of the skin [24]. It seems that the main factor triggering these lesions is improper resting surfaces because these injuries were identified even in obese horses, which had no other body lesions [23]. The results of the present study seem to confirm this assumption, as the prevalence of hip point lesions decreased significantly after the stallions had access to softer and less abrasive resting surfaces of their choice outside the barn (Table 3). 

#### 4.1.3. Freedom from Pain, Injury, and Disease

Most health problems were more common in the tie-stalled stallions, and many of these improved significantly in time after their transition to free group housing (Table 3). For example, the abnormal hair coat condition noticed in eight stallions in the first assessment persisted in only one of them until the final (third) evaluation, a significant (*p* < 0.05) improvement (Table 3). Body condition and coat quality have been widely used indicators in welfare assessment protocols (e.g., the Working Equine Welfare Assessment protocol or Standardized Equine Based Welfare Assessment tool—SEWBAT [25] and Animal Welfare Indicators—AWIN [16] protocols), both in working and recreational horses [26]. As for body lesions, their prevalence increased significantly after the stallions’ transition to free group housing (from the first to the second assessment—A1 to A2), and then it decreased from the second to the third assessment. As opposed to several studies reviewed by Gehlen et al. [5] on adult stallions’ transition from isolated to free group housing, minimal previous acclimatization was provided to the animals in the present study. As stated above, we do not encourage the described conditions to be reproduced, especially without continuous and close monitoring of the stallions. The owner’s choices and decisions nevertheless allowed us to observe the consequences of this type of transition. The large open space enabled escape possibilities for the animals from each other, a very important positive aspect, but, on the negative side, the lack of more diverse previous social contact triggered aggressive encounters, especially in the first few days after their release. Group dynamics can change rapidly in this type of setting. As Briefer Freymond et al. [27] describe, ritual and affiliative interactions do not involve physical aggression, but agonistic interactions have the potential to do so, and all three occur within a group of free stallions. In only three to four days, the frequency of agonistic and ritual behaviors decreases in the group, according to Briefer Freymond et al. [27], and certain previous experiences in group housing may minimize aggression between free-housed stallions (when their management is periodically switched between individual and group housing). In our opinion, the visual, olfactory, and auditive contacts possible between the studied stallions led to a certain degree of acclimatization, even if each animal could best experience these with only two neighbors (both while tethered inside the barn and outside in the paddocks), due to the farm’s daily routines. These practices were meant to minimize aggression in the given setting but rotating the stallions without being allowed tactile interaction could have been beneficial at the moment of their release (a higher number of horses acclimatized with each other previously). Furthermore, closer social contact, with the possibility of tactile interaction, such as the use of “social boxes” [5], could have lowered aggression during the stallions’ group integration. However, after the initial increase in the frequency of body lesions (recorded two weeks after the housing system change), the number of deep wounds decreased significantly (*p* = 0.001) to none in three months, even if the frequency of superficial body lesions remained five times higher than before the management system change. According to Briefer Freymond et al. [27], a stable hierarchy is established and measurable in two to three months after the stallions are released as a group. This is consistent with our results regarding the decreasing frequency of more serious (deep) wounds, mainly produced by aggressive encounters between the stallions on the first days of free group housing. The high prevalence of superficial body lesions recorded at three months from the management change included several wounds which have been found to be deep at the previous welfare assessment but healed meanwhile. 

Some of the health parameters of freedom from pain, injury, and disease improved as a consequence of the management change but not of the group housing in itself. The significantly lower prevalence (*p* < 0.05, Table 3) of lip corners, harness contact points, and feet lesions occurred through the healing of previous wounds and non-exposure to new ones, as the stallions have not been used for work at all during the study. The improvement of their hoof horn quality may have been related to nutrition with higher biotin and vitamin quantities (grass vs. hay), although for a pertinent conclusion, a longer study period would be needed. As Josseck et al. [28] describe, biotin supplementation does not accelerate hoof growth (7 mm/28 days, complete hoof renewal in 11 months), but it leads to significant hoof condition improvement in nine months; only a third of that period was covered by the present study, and our differences were not statistically significant (*p* > 0.05, Table 3). 

The condition of more than half of the horses with swollen tendons/joints improved in only two weeks, and 82% of those with leg swellings at the first assessment were free of this problem in three months (Table 3); the difference between successive assessments being highly significant (*p* < 0.001). Although no diagnostic was established for the volume and shape modifications of the legs, based on their quick and spontaneous remedial (without any treatment or movement restrictions), the cause might have been mainly circulatory (peripheral edemas) due to insufficient exercise for some of the stabled stallions. However, volume changes in horse limbs were shown to occur quite quickly in relation to exercise. Using an optometric device, Siewert et al. [29] measured a significant decrease in limb volume immediately after exercise (compared to the inactive value measured after a 12 h standing period) but also an increase after one hour of rest following the exercise, with significantly bigger changes in male horses compared to females. Thus, in our study, the edematous limb swellings could have also been caused by excessive effort (in the stallions used for wood-hauling). For possibly similar reasons, the lameness prevalence decreased between successive assessments, but the number of stallions with abnormal gait did not. 

Respiratory problems (dyspnea, cough, nasal discharge) had a higher frequency in our first assessment while the stallions were tie-stall housed later (Table 3) while kept outside. It was previously found that poor indoor air quality can negatively impact the respiratory health of both horses and humans [30]. The tight link between equine respiratory health and air quality was recently reinforced once again through an interdisciplinary effort over 19 years (between 2000 and 2019) to study equine airway inflammation, to clarify the phenotype and terminology involved, and to finally introduce the term ‘equine asthma’ for horses with chronic respiratory signs, previously referred to as ‘inflammatory airway disease’ and ‘recurrent airway obstruction’. The 2019 report [31] on this topic states that the role of exposure to environmental dust in the pathophysiology of both mild and severe equine asthma is supported by strong evidence. Thus, the conclusion that “the pasture is the best housing” drawn more than 20 years ago by McGorum et al. [32] still holds ground. 

#### 4.1.4. Freedom to Express Normal Behavior

In addition to better air quality, group housing on a pasture has tremendous benefits regarding the freedom to express normal behavior. As expected, compared to the previous (tie-stall) management system in the present study, both parameters (the company of other horses and access to free exercise, Table 3) showed statistically significant differences. Daily free-running is a behavioral and physiological requirement for all horses [33] as neither work nor the use of training devices can fulfill their need for free exercise [34]. In addition to the general health-preserving benefits of free exercise on the respiratory tract, locomotor apparatus, and immune system of horses, and against the traditional misbelief that performing horses need rest rather than free movement [35], the positive consequences of active recovery have been shown not only in terms of recovery time and performance but also of general welfare improvements, even in horses on intensive physical effort programs [33]. 

In respect of the importance of company of the same species Dierendonck [36] concludes in the light of the large amount of available literature that social positive physical interactions (allogrooming, play) represent an ethological need for horses, a highly motivated behavior, so important for the animal that husbandry systems that do not provide access to it cause chronic stress. The author states that all horses need physical and social contact [36]. Although with a special focus on horse–human interactions, Rørvang et al. [37] describe the importance of each sensory ability in equine communication with individuals of the same or different species, highlighting the possible reinforcing value of tactile stimulation during affiliative interactions (mutual grooming, swishing flies for one another, and standing in close proximity while grazing or resting). These findings indicate that free interactions between horses are much better than olfactory, visual, and auditive interactions in stabled or individually exercised animals. Of course, limited interaction with their own kind was proved to still be better than the lack of it (complete isolation), as Houpt and Waran [38] admit for mares chronically confined in tie-stalls with severe movement restrictions (in the pregnant mare urine production industry) developing fewer stereotypies compared to box-stalled Thoroughbreds. 

#### 4.1.5. Freedom from Fear and Distress

This section of the welfare assessment explored the dynamics of the horse–human relationship during the management transition. Our results did not reveal any significant impact of the pasture release on the stallions’ responsivity toward humans (the owner or caregivers). However, a reduction of the indifference to humans can be observed (Table 3) when the results of the first assessment were compared to those of the third (alongside the increasing welfare degree of the stallions). Some authors [39,40] found the strongest correlations between indifference and the absence of free exercise, barn dirtiness, poor body condition, and health problems in working equines. 

The ability of horses to recognize familiar humans (based on a global, integrated, multisensorial representation of the person) and to adapt their behavior to expectancies (based on previous experiences) in a familiar situation with that person has been proven by several studies [41,42]. Another important scientific finding along the same lines was the first-time discovery that horses are capable of spontaneous cross-modal recognition of individuals from a morphologically very different (and phylogenetically very distant) species, matching visual and auditory information from familiar humans [43]. More recent research revealed their ability to differentiate between familiar and unfamiliar humans from photographs of faces [44] and the fact that they employ a holistic mental approach rather than processing the images as simple abstract shapes [40], clarifying even more that horses recognize and remember their caregivers, even when they have no interaction with them for longer periods of time [45]. Our results regarding the response of the stallions at the approach, walking beside, and touch of the familiar person (Table 3) are explained by this equine ability to recognize and remember a human, and also to adapt their behavioral reactions according to their previous experiences—neutral or positive in the present study. As Boissy et al. [46] proved, positive interactions and experiences with humans have beneficial effects on improving the animals’ welfare degree, and, we could add, the effects persist even through management changes which provide more freedom (and avoidance possibility) to the stallions in our case. 

### 4.2. Effects of the Management Change on the Stallions’ Overall Welfare and Docility

Changing the housing and management system from tie-stalls with limited outside access and social contact to free group housing had a positive influence on the studied stallions’ overall welfare, their individual welfare quality scores (IWQS) being significantly higher in the second and third assessments than in the first one (Table 4, Figure 1). The overall improvement in the stallions’ welfare was also visible when they were assigned to welfare categories based on their IWQS in each of the three assessments (Figure 2). This finding is in accordance with other studies which show that living in a paddock or on a pasture is more appropriate than living in a box for the welfare of stallions [4,11,13]. Providing horses with a living environment more similar to their natural conditions is part of welfare improvement [13], especially when the simultaneous contribution to each of the five freedoms are considered. However, certain elements of the environment and management system influence several aspects of the overall welfare, being framed in more than one freedom. The free group housing on a pasture compared to the tie-stall management improved at the same time the physical, mental, and natural components of the stallions’ welfare, bringing measurable benefits to each freedom. Under natural conditions, horses spend between 75% and 90% of their time grazing [10,47], usually walking continuously at a slow pace [48]. According to McGreevy [49], this way, they can travel as much as 65–80 km per day, having all the benefits of the social interactions in the group. After the management change, the studied stallions had constant access to pasture and a water source, a clean and comfortable surface to rest on—the soft, warm, and dry earth of the pasture under the protection of dense tree canopy—access to the company of the same species, being allowed natural behaviors such as grazing, running, and rolling, and not being frightened and stressed by human interventions. These conditions fulfilled four out of the five freedoms of animal welfare. The only less addressed freedom was the one from pain, injury, and disease, although frequent monitoring and surveillance enabled timely intervention and treatment when required, even prevention at all times. In the present study, an improvement in the stallions’ welfare quality was observed in as little as two weeks from their transition to free group housing. This proves a positive influence of the free system on the overall welfare of the horses, an aspect that has been previously suggested but not proved by other studies which focused only on selected elements (health, behavior, human–horse relationship, and so on). 

One of the widespread concerns about horses kept with less frequent human interactions is whether they maintain (and for how long) their willingness to remain ‘submissive’ to humans or if their docility decreases when handled less, and there is not much systematical research available to answer these questions. The timing of the decrease in the frequency of human manipulation, how well the previous training has been conducted, and for how long no element of it is recalled are all relevant factors. However, with regard to human safety around horses, Rivera et al. [12] noted that horses housed in paddocks are less aggressive toward humans than those kept in boxes. The importance of docility has been recognized in horses since the early times of domestication and selective breeding and later on, during the development of different classical horse breeds [50]. Still highly desirable in the entire horse industry, docility is continuously studied today. As recent genetic sequencing research [51] shows, through the discovery of a genomic region that may have influenced it, docility and a strong back were even highly valued traits in the Bronze Age. Other studies, using the term trainability for docility, attempted to determine a possible connection between this trait and hormones such as oxytocin and serotonin [52,53]. In our study, the housing system’s change did not influence the docility score of the stallions. Given the numerous studies proving that horses are able to recognize humans [41,42,43,44,45] and remember familiar persons for longer periods of time even without seeing them [45], the plausible explanation of this finding was the fact that the people interacting with the stallions were the same with whom they had been habituated previously. 

### 4.3. Effects of the Management Change on the Qualitative Behavioral Assessment (QBA) Descriptors of the Studied Stallions

Although no significant differences were found regarding the qualitative behavioral assessment (QBA) of the stallions in the successive evaluations (transition from one management system to another, time passed since the management change), some aspects were still notable. As Table 6 and Table 7 show, considering only the variables for which the correlation coefficients between the original variables and PCs were above 0.2 (as recommended by Bassler et al. [17]), the stallions were more annoyed, apathetic, uneasy, pushy, and less at ease and happy, than later. In the second and third evaluations, the animals seemed happier and less apathetic than during the first determination. As their welfare improved, the stallions seemed to be happier. These findings support the statement of Boissy et al. [46] that if an animal is in a happy emotional state, then its needs are being met, and its welfare is good. 

The QBA was developed and promoted to determine and include indicators of the animals’ positive state in welfare assessments. An intrinsically holistic and dynamic tool, QBA addresses the whole animal in terms of its behavior [54], allowing the assessor to integrate these behavioral expressions using descriptors that reflect the animal’s putative emotional experiences [55]. Applied in an impressive number of studies on many domestic animal species, QBA was even proposed as a potential ‘first pass’ screening method to decide if a further in-depth assessment may be warranted on a specific farm [56]. Far from being generally accepted as an “ideal method”, QBA was also firmly disputed in the scientific literature for its limitations [57,58].

Specifically in horses, QBA was found to be useful in identifying more positive affective states [54]. Indeed, irrespective of the assessment in our study (before the management change, two weeks later, or three months later), the tendencies of almost all principal components (PCs) showed a direction from negative to positive emotional states, except for the first assessment, where on PC2 the tendency was from pushy to apathetic. As the overall welfare of the stallions improved, the tendency on PC1 became richer (Table 5), and the stallions added progressively to the friendly and relaxed attitudes (first assessment), curiosity (in the second assessment), and then a keenness for contact (look for contact, in the third assessment). Less relevant, on PC2 in the second and third assessments, the tendency gravitated around aggressiveness and a pushy attitude (defined as assertive or forceful, not leaving space, head butting, exhibiting dominant behavior, possibly mouthy or nippy [AWIN, 2015]), with a rather negative connotation. On PC3, the tendency was constant, from apathetic to happy, irrespective of the housing system and conditions the stallions were in. Analyzing these results, we note that the QBA identified positive states in each assessment of the studied stallions, but their management transitioning and change in their overall welfare were not closely reflected by the dynamics of the emotional states identified. 

Moreover, when looking at the relationship between the PCs and the original variables (Table 6), a constant considerable relationship (with a correlation coefficient above 0.2 as recommended by Bassler et al. [17]) was found only for apathy and happiness on PC3, and for pushy attitude on PC2 and PC3, with the strongest correlations for the first two (apathy and happiness), lessening the importance of the other 10 descriptors (Table 6) for our study. As Hausberger et al. [59] note in a comprehensive review of equine welfare assessment methods, the validity of QBA remains in question. The same review shows that this method’s validation has not been tested for adult horses. The same authors [59] warn that human representations of behaviors’ significance may be influenced by a variety of factors (culture, access to a reference population, personal experience), which can introduce a bias-induced error at the very early stages of data collection. 

### 4.4. Interrelations between the Studied Parameters during the Stallions’ Management Change

To explore the significance of the QBA descriptors in the specific setting in our study further, the PCs were studied in relation to the IWQS and DS of the stallions (Table 7). When it comes to the statistically significant correlations between QBA scores and physiological or other quantitative measures relevant to welfare proved by many authors (reviewed by Fleming et al. [56]), not all research performed is congruent. Andreasen et al. [60] show weak correlations of QBA scores to Welfare Quality^®^ measures (and no meaningful pattern of relationship between these measures) in dairy cows. Similarly, Hausberger et al. [59] note that the relationship between welfare indicators and QBA results is not straightforward for donkeys. In our study, the correlations were found to be inconsistent: the PC1 correlated significantly with the IWQS in the first (A1) and third (A3) assessments; the PC3 correlated with the IWQS and DS in the first assessment (A1), and in the second assessment (A2) only with the DS (Table 7). 

Although QBA is a promising tool to complement horse welfare assessments in situations of multiple emotional dimensions of both positive and negative valences [55], and it is sensitive to the quality of the horse–human relationship [54], in light of our results of weak correlations and low relevance for the assessment for the stallions’ management transition, we consider that most QBA descriptors did not fit the type of study described in this paper. More specific research in similar conditions is required.

Regarding the relationship between the IWQS of the stallions and their DS, interestingly, these showed constantly significant positive correlations in all three assessments, enforcing the hypothesis that the docility of the stallions did not decrease as their welfare improved, even if they had been handled by humans less frequently during the period of our study. 

## 5. Conclusions

Given the above results, we conclude that the free group housing of adult stallions improved their overall welfare and did not reduce their docility toward familiar humans compared to their previous management system (tie-stall housing). These are two important aspects that have not been studied in this manner before. The successive assessments showed the improvement of the stallions’ IWQS in only two weeks from the change in the management system, with further improvements in time once the group’s hierarchy stabilized. The constant positive correlation between the DS and IWQS of the stallions in the successive assessments (despite less frequent human–animal contact after the management change) is a promising result, further providing incentives to stallion owners who consider implementing similar management changes for their animals. Many of the assessed qualitative behavioral descriptors showed no relevance for our study and followed neither the welfare degree nor the docility level of the stallions closely. Thus, we consider that most QBA descriptors require more specific studies in similar conditions to those described. 

## Figures and Tables

**Table 1:** Parameters and scoring in the welfare assessment of the 32 studied stallions.

No	Parameter	Assessment Description
Freedom from hunger and thirst		
1	Body Condition Score (BCS)	Visual and palpatory, on the 5-point scale [15] and scored as 0—improper BCS (emaciated, thin, fat, and obese conditions); 1—good body condition
2	Water cleanliness	Visual, a filled 2 L transparent glass bottle assessed for any change in color and/or turbidity compared with drinking water (0—dirty; 1—clean)
Freedom from discomfort		
3	Body soiling	Visual, assessing the haircoat for any foreign material sticking, which covers an area bigger than the palm of a hand (0—present; 1—absent)
4	Hip point lesions	Visual, assessed on both sides (0—skin lesion; 1—scar, thickened skin with alopecia; 2—absent)
Freedom from pain, injury, or disease		
5	Hair coat condition	Visual, assessed to identify dull, matted hair with or without skin debris and/or alopecia (0—abnormal on extended areas, more than 20 cm in diameter, the presence of several small alopecic spots included; 1—abnormal on limited areas, less than 20 cm in diameter; 2—normal)
6	Hair quality in the mane/tail	Visual, assessed to identify missing and/or broken hairs, skin debris, and dull hair (0—abnormal; 1—normal)
7	Body lesions	Visual and palpatory, assessed to identify interruptions in skin integrity, except for the feet (0—deep lesions interrupting at least the whole thickness of the skin; 1—superficial lesions, without complete penetration of the skin; 2—the absence of lesions)
8	Feet lesions	Visual and palpatory, assessed below the knees and hocks (0—deep lesions interrupting at least the whole thickness of the skin; 1—superficial lesions, without complete penetration of the skin; 2—the absence of lesions)
9	Lip corner lesions	Visual and palpatory, assessed for any visible lesion (0—at least one lesion; 1—the absence of lesions)
10	Lesions at harness contact points	Visual and palpatory, assessed in the body areas where the specific harness would be in contact with the body (0—at least one disruption of the skin integrity; 1—missing hair at the harness contact points with no skin interruption; 2—the absence of lesions)
11	Swollen tendons/joints	Visual, assessing the legs and feet (0—both tendons and joints swollen in at least one area; 1—at least a tendon or joint swollen; 2—the absence of swellings)
12	Hoof horn quality	Visual, looking from above, without uplifting the feet, previously washed as needed (0—abnormal, with interruptions, rough surface, lacking periople; 1—normal hoof horn)
13	Hoof shape	Visual, as above (0—abnormal including all possible deviations of shape; 1—normal)
14	Gait	Visual, assessed in the horse walked for at least 10 m on even terrain and turned in both directions (0—lame; 1—abnormal gait; 2—sound)
15	Dyspnea	Visual, assessing the nostrils (straight from the front) and lateral body areas (at an angle of 45° from behind) (0—present; 1—absent)
16	Cough	Auditory, assessed to record any cough over the whole assessment (0—present; 1—absent)
17	Nasal discharge	Visually assessed during the dyspnea assessment (0—present; 1—absent)
18	Ocular discharge	Visual (0—presence of mucopurulent or purulent ocular discharge;1—presence of serous ocular discharge; 2—absence of ocular discharge)
19	Diarrhea	Visual, assessed on the medial and/or caudal aspect of the hindlegs for any fecal soiling (0—present; 1—absent)
Freedom to express normal behavior		
20	Company of other horses	Visual (0—none; 1—limited; 2—unrestricted)
21	Access to free exercise	Visual and owner declaration, assessing the possibility of free exercise in a space that allows minimum 5 steps at gallop in minimum 2 directions for a minimum of 1 h per day (0—none; 1—limited; 2—unrestricted)
Freedom from fear and distress		
22	General alertness	Visual, assessed by observing the horse’s body position and response to environmental stimuli (0—apathetic/depressed; 1—alert)
23	Response to the familiar person approaching	Visual, assessed by observing the horse’s body language (0—aggressiveness; 1—fear/avoidance; 2—indifference; 3—friendliness)
24	Response to the familiar person walking beside	Visual, assessed by observing the horse’s body language (0—aggressiveness; 1—fear/avoidance; 2—indifference; 3—friendliness)
25	Response to the familiar person touching	Visual, assessed by observing the horse’s body language (0—aggressiveness; 1—fear/avoidance; 2—indifference; 3—friendliness)

**Table 2:** Parameters and scoring in the docility assessment of the 32 studied stallions.

	Docility	
1	Response to the familiar person putting a rope around the base of the horse’s neck	Visual, assessed by observing the horse’s body language (0—aggressiveness; 1—fear/avoidance; 2—indifference; 3—friendliness)
2	Response to the familiar person attaching a halter on the horse’s head	Visual, assessed by observing the horse’s body language (0—aggressiveness; 1—fear/avoidance; 2—indifference; 3—friendliness)

**Table 3:** Results for the welfare parameters (improper) of the stallions in each of the three assessments and the significance of differences between them.

Parameter	Percentage of Stallions (Number of Stallions)			*p* Value (Friedman Test)
	A1	A2	A3	
Freedom from hunger and thirst				
BCS (improper)	21.88 (7)	25 (8)	12.5 (4)	0.197
Water cleanliness (dirty)	21.88 (7)	0	0	0.001
Freedom from discomfort				
Body soiling (present)	31.25 (10)	12.5 (4)	18.75 (6)	0.135
Hip point lesions (present)	34.38 (11)	25 (8)	3.13 (1)	<0.001
Freedom from pain, injury, and disease				
Hair coat condition (abnormal)	25 (8)	15.63 (5)	3.13 (1)	0.008
Hair quality in the mane/tail (abnormal)	3.13 (1)	9.38 (3)	9.38 (3)	0.368
Body lesions (present)				0.003
- deep	6.25 (2)	15.63 (5)	0	0.05
- superficial	6.25 (2)	34.38 (11)	31.25 (10)	0.016
Feet lesions (present)				0.001
- deep	9.38 (3)	3.13 (1)	0	0.164
- superficial	18.75 (6)	15.63 (5)	0	0.043
Lip corner lesions (present)	15.63 (5)	3.13 (1)	0	0.015
Lesions at harness contact points (present)	28.13 (9)	25 (8)	6.25 (2)	0.001
Swollen tendons/joints (present)	34.38 (11)	15.63 (5)	6.25 (2)	<0.001
Hoof horn quality (abnormal)	9.38 (3)	6.25 (2)	3.13 (1)	0.135
Hoof shape (abnormal)	12.5 (4)	12.5 (4)	9.38 (3)	0.368
Gait				0.045
- lame	12.5 (4)	9.38 (3)	0	0.05
- abnormal	18.75 (6)	25 (8)	21.88 (7)	0.834
Dyspnea (present)	12.5 (4)	6.25 (2)	0	0.05
Cough (present)	9.38 (3)	6.25 (2)	0	0.174
Nasal discharge (present)	15.63 (5)	6.25 (2)	0	0.004
Ocular discharge (present)	12.5 (4)	15.63 (5)	3.13 (1)	0.074
Diarrhea (present)	0	15.63 (5)	0	0.007
Freedom to express normal behavior				
Company of other horses				<0.001
- none	0	0	0	<0001
- limited	100 (32)	0	0	<0.001
Access to free exercise				<0.001
- none	71.88 (23)	0	0	<0.001
- limited	28.13 (9)	0	0	<0.001
Freedom from fear and distress				
General alertness (apathetic/depressed)	6.25 (2)	0	0	0.368
Response to the familiar person approaching				0.368
- aggressiveness	0	0	0	-
- fear/avoidance	3.13 (1)	0	3.13 (1)	0.603
- indifference	31.25 (10)	43.75 (14)	28.13 (9)	0.383
- friendliness	65.63 (21)	56.25 (18)	68.75 (22)	0.561
Response to the familiar person walking beside				0.819
- aggressiveness	0	0	0	-
- fear/avoidance	6.25 (2)	6.25 (2)	3.13 (1)	0.812
- indifference	43.75 (14)	40.63 (13)	37.5 (12)	0.802
- friendliness	50.00 (16)	53.13 (17)	59.38 (19)	0.748
Response to the familiar person touching				0.595
- aggressiveness	0	0	0	-
- fear/avoidance	6.25 (2)	6.25 (2)	9.38 (3)	0.859
- indifference	62.5 (20)	34.38 (11)	37.5 (12)	0.068
- friendliness	31.25 (10)	59.38 (19)	53.13 (17)	0.063

A1—first assessment; A2—second assessment; A3—third assessment. If the *p*-value is less than 0.05, the difference between assessments is significant.

**Table 4:** Descriptive statistics of the individual welfare quality and docility scores in the assessed stallions in each of the three assessments performed.

Parameter	Individual Welfare Quality Scores (IWQS)			Individual Docility Scores (IDS)		
	A1	A2	A3	A1	A2	A3
Mean	32.28	36.03	38.41	4.71	4.50	4.47
Standard Error of the mean	0.72	0.54	0.36	0.20	0.21	0.21
Median	33.5 ^a^	36.00 ^b^	38.5 ^c^	5.00	4.50	5.00
Minimum	24.00	31.00	33.00	2.00	2.00	2.00
Maximum	37.00	41.00	41.00	6.00	6.00	6.00

^a,b,c^ Values in a row with no common superscript are significantly different (*p* < 0.05).

**Table 5:** Loadings for the QBA descriptors on the first three principal components.

Descriptor	A1			A2			A3		
	PC1	PC2	PC3	PC1	PC2	PC3	PC1	PC2	PC3
Aggressive	−0.183	0.190	-	-	0.721 *	-	-	0.694 *	0.130
Alarmed	−0.779 *	-	−0.149	−0.781 *	0.168	−0.307	−0.862 *	-	−0.245
Annoyed	−0.526 *	0.646 *	0.104	−0.471 *	0.605 *	0.132	−0.607 *	0.608 *	-
Apathetic	−0.170	−0.325	**0**.861 *	−0.273	−0.379	−0.833 *	−0.134	−0.455 *	−0.815 *
At ease	0.593 *	−0.380 *	0.433 *	0.559 *	−0.394	0.321	**0**.737 *	−0.354	0.129
Curious	**0**.664 *	0.389	-	0.717 *	0.462 *	-	0.815 *	0.447 *	-
Friendly	**0**.842 *	0.283	-	**0**.886 *	0.259	0.129	**0**.879 *	0.269	-
Fearful	−0.896 *	-	−0.250	−0.824 *	-	−0.357	−0.910 *	-	−0.276
Happy	−0.627 *	-	−0.589 *	0.637 *	0.135	0.628 *	0.445 *	0.277	**0**.731 *
Look for contact	**0**.595 *	0.213	-	0.731 *	0.495 *	-	0.724 *	0.487 *	-
Relaxed	**0**.791 *	−0.272	−0.252	0.720 *	−0.413 *	−0.210	**0**.757 *	−0.326	−0.210
Pushy	0.311	**0**.659 *	0.344	0.402	**0**.627 *	0.354	0.318	**0**.736 *	0.348
Uneasy	−0.534 *	0.641 *	-	−0.546 *	0.630 *	0.257	−0.651 *	0.602 *	0.189
Eigen values	5.005	2.914	1.575	5.117	2.722	1.695	5.761	2.784	1.585
Variance explained (%)	38.501	22.418	12.112	39.364	20.938	13.040	44.313	21.417	12.193
Cumulative variance explained	38.501	60.919	73.030	39.364	60.302	73.342	44.313	65.730	77.923

The numbers with asterisks (*) indicate higher (positive or negative) loadings in the three assessments within each PC.

**Table 6:** Component score coefficient matrix for three principal components in the three qualitative behavior assessments of the stallions.

Descriptor	A1			A2			A3		
	PC1	PC2	PC3	PC1	PC2	PC3	PC1	PC2	PC3
Aggressive	−0.03	0.17	−0.01	−0.01	0.26 *	0.01	−0.01	0.25 *	0.08
Alarmed	−0.15	0.02	−0.09	−0.15	0.06	−0.18	−0.15	0.02	−0.15
Annoyed	−0.10	0.22 *	0.07	−0.09	0.22 *	0.08	−0.10	0.22 *	0.05
Apathetic	−0.03	−0.11	0.55 *	−0.05	−0.14	0.49 *	−0.02	−0.16	0.51 *
At ease	0.12	−0.13	0.27 *	0.10	−0.15	0.19	0.13	−0.13	0.08
Curious	0.13	0.20 *	0.06	0.14	0.17	0.05	0.14	0.16	0.02
Friendly	0.17	0.10	0.04	0.17	0.09	0.08	0.15	0.11	0.02
Fearful	−0.18	−0.02	−0.16	−0.16	0.03	−0.21 *	−0.16	0.01	−0.17
Happy	0.13	0.03	−0.37 *	0.12	0.05	−0.37 *	0.08	0.10	0.46 *
Look for contact	0.12	0.18	0.04	0.14	0.18	−0.04	0.13	0.18	0.05
Relaxed	0.16	−0.09	−0.16	0.14	−0.15	−0.12	0.13	−0.12	−0.13
Pushy	0.06	0.23 *	0.22 *	0.08	0.23 *	0.21 *	0.05	0.26 *	0.22 *
Uneasy	−0.11	0.22 *	0.06	−0.11	0.23 *	0.15	−0.11	0.22 *	0.12

Extraction method: Principal component analysis; * coefficients beyond 0.2 reflect a considerable positive/negative relationship between the principal component and the original variable [17].

**Table 7:** Correlations between the first three principal components (PCs) and the IWQS and DS in the studied stallions.

Assessment	A1		A2		A3	
Principal Component	IWQS	DS	IWQS	DS	IWQS	DS
PC1	0.35 *	0.32	0.33	0.19	0.36 *	0.32
PC2	0.05	0.18	0.11	0.11	0.14	−0.09
PC3	0.48 *	0.38 *	−0.25	0.43 *	0.00	0.24

* Correlation is significant at the 0.05 level (2-tailed).

## Data Availability

Not applicable.
